# Does Fluctuating Light Affect Crop Yield? A Focus on the Dynamic Photosynthesis of Two Soybean Varieties

**DOI:** 10.3389/fpls.2022.862275

**Published:** 2022-04-25

**Authors:** Nicole Salvatori, Giorgio Alberti, Onno Muller, Alessandro Peressotti

**Affiliations:** ^1^Department of Life Sciences, University of Trieste, Trieste, Italy; ^2^Department of Agricultural, Food, Environmental, and Animal Sciences, University of Udine, Udine, Italy; ^3^Faculty of Science and Technology, Free University of Bolzano, South Tyrol, Italy; ^4^Institute of Bio- and Geosciences Plant Sciences (IBG-2), Forschungszentrum Jülich GmbH, Jülich, Germany

**Keywords:** dynamic photosynthesis, growth chamber, fluctuations, gas exchange, NPP, chlorophyll deficient

## Abstract

In natural environments, plants are exposed to variable light conditions, but photosynthesis has been mainly studied at steady state and this might overestimate carbon (C) uptake at the canopy scale. To better elucidate the role of light fluctuations on canopy photosynthesis, we investigated how the chlorophyll content, and therefore the different absorbance of light, would affect the quantum yield in fluctuating light conditions. For this purpose, we grew a commercial variety (Eiko) and a chlorophyll deficient mutant (MinnGold) either in fluctuating (F) or non-fluctuating (NF) light conditions with sinusoidal changes in irradiance. Two different light treatments were also applied: a low light treatment (LL; max 650 μmol m^−2^ s^−1^) and a high light treatment (HL; max 1,000 μmol m^−2^ s^−1^). Canopy gas exchanges were continuously measured throughout the experiment. We found no differences in C uptake in LL treatment, either under F or NF. Light fluctuations were instead detrimental for the chlorophyll deficient mutant in HL conditions only, while the green variety seemed to be well-adapted to them. Varieties adapted to fluctuating light might be identified to target the molecular mechanisms responsible for such adaptations.

## Introduction

Photosynthesis has been extensively studied since the beginning of the 20th century (Stirbet et al., 2019) also to improve it through genetic engineering with the aim of increasing yield potential of crops (Sinclair et al., 2019; Yoon et al., 2020). However, most of the studies and models on photosynthesis have considered steady-state and/or extremely controlled conditions (Rascher and Nedbal, 2006). This has facilitated the reliability and reproducibility of the data and the elaboration of important conceptual frameworks, but the results of these studies are referring to situations far from the ones occurring in natural environments (Matsubara, 2018). In particular, one of the most variable conditions is the incident light (Pearcy, 1990), which forces the processes of photosynthesis to continuously change to optimize the use of the incoming energy and to avoid photo-inhibition (Kaiser et al., 2018a). The understanding of the mechanisms of adaptations to such dynamic conditions is fundamental to improve photosynthesis in the field and enhance the overall crop yield (Foyer et al., 2017), by reducing the possible limiting processes (Taylor and Long, 2017) or by selecting or creating varieties well-adapted to fluctuating light environments (Alter et al., 2012; Acevedo-Siaca et al., 2020; Kaiser et al., 2020).

The understanding of the general effect of fluctuating light on photosynthesis (the so-called “dynamic photosynthesis”) and on the overall carbon (C) uptake by plants is complicated by the fact that the effect has been evaluated on different species measured under different growth conditions. Several studies at leaf scale have proposed that light fluctuations may decrease the daily integral C assimilation (e.g., Morales and Kaiser, 2020), whereas others (e.g., Graham et al., 2017) have suggested that specific fluctuations periods and intensities might even enhance photosynthesis. Fluctuations of light force plants to constantly be in unsteady-state conditions and their ability to promptly respond to such variations mainly depends on the efficiency of antenna complexes (Kaiser et al., 2018a). The light-harvesting complex II (LHCII) mainly regulates the energy distribution between PSI and PSII and the thermal dissipation of excess absorbed energy (Horton et al., 1996; Flexas et al., 2012). In order to investigate proper strategies to improve dynamic photosynthesis and increase plant production, Kaiser et al. (2019) have proposed three main possible approaches to enhance dynamic photosynthesis: (i) the acceleration of relaxation rates of photoprotection; (ii) the acceleration of Calvin–Benson–Bassham enzyme activation/deactivation; (iii) the acceleration of stomatal dynamics. However, experiments have given controversial results. For example, as far as the first approach is concerned, Kromdijk et al. (2016) reported an increase in C uptake in tobacco plants under fluctuating light by stimulating a faster onset of photoprotection. On the contrary, Garcia-Molina and Leister (2020) found a decrease in biomass accumulation after applying the same approach in Arabidopsis. However, most of the abovementioned studies have been performed at the leaf level and/or on plants grown under homogeneous light conditions and then suddenly exposed to fluctuating light (Marler, 2020). Thus, even though they have been fundamental to unravel the molecular mechanisms of photosynthesis and possible strategies to improve it under fluctuating light, they can be hardly scaled up to the entire canopy (Matsubara, 2018). Therefore, it is fundamental to focus on what it is happening at canopy level under natural fluctuating environmental conditions. Only more recently, some studies have been simulating natural light conditions at canopy level by either artificial fluctuations (i.e., alternating low and high light, Suorsa et al. (2012); short light flecks, Kaiser et al. (2018a); sinusoidal variations of light, Matthews et al. (2018)) or by simulating natural occurring light fluctuations (Vialet-Chabrand et al., 2017a; Ferroni et al., 2020), but more experiments are needed in this direction. Moreover, the understanding of the mechanisms behind dynamic photosynthesis is also limited by some technical constraints. Canopy gas exchange measurements can be based on the use of micro-meteorological techniques (i.e., eddy covariance) or remote sensing (i.e., chlorophyll fluorescence). All these methods can give interesting results, but they can suffer of some weaknesses. For example, specific environmental conditions are needed for eddy covariance to obtain reliable data (Wang et al., 2010), whereas remote sensing approaches still necessitate a validation with C uptake measurements (Mohammed et al., 2019). On the other hand, growth chambers (Wang et al., 2018) and/or ecotrons (Roy et al., 2021) have become standard tools to simulate different environmental conditions and disentangle their influences on the canopy or even ecosystem functioning.

To better elucidate the role of light fluctuations on canopy photosynthesis, we investigated how the chlorophyll content, and therefore the different absorbance of light, would affect the quantum yield in fluctuating light conditions. We hypothesized that in fluctuating light conditions leaves with less chlorophyll would undergo smaller changes in incident light intensities (simply by absorbing less light) and consequently would stress less the photoprotective machinery, which is thought to interfere with the velocity of photochemical quenching induction/relaxation dynamics in fluctuating conditions (Kromdijk et al., 2016). To test this hypothesis, we assessed the response of two soybean cultivars grown in controlled conditions (Salvatori et al., 2021) and exposed to different light conditions (constant and fluctuating light): a green cultivar (Eiko) and a chlorophyll deficient mutant (MinnGold). In particular, we tested if MinnGold and Eiko showed the same net primary production (NPP) at canopy scale under non-fluctuating light and if Eiko showed a more pronounced decrease in NPP under fluctuating light.

## Materials and Methods

### Plant Materials and Growth Conditions

We grew a commercial green soybean variety (Eiko, Asgrow, United States) and the chlorophyll deficient mutant MinnGold (Campbell et al., 2015) in a growth chamber system (DYNAMISM; Salvatori et al., 2021). MinnGold is the result of a non-synonymous substitution in a magnesium chelatase ChlI subunit leading to plants with a “yellow” or “golden” phenotype with approximately 80% less chlorophyll than the green varieties (Campbell et al., 2015; Sakowska et al., 2018). DYNAMISM is composed of twelve 0.54 m^3^ open-top growth chambers, equipped with a dimmable LED system, for instantaneous net canopy CO_2_ flux (A; μmol CO_2_ m^−2^ s^−1^) and evapotranspiration (E; mol H_2_O m^−2^ s^−1^) measurements at canopy scale. Plants were sown in pots (13 × 13 × 18 cm) with siliceous sand thus to have an inert substrate without any microbial contribution to instantaneous CO_2_ fluxes (heterotrophic respiration = 0).

Two separate experiments were done to assess the different responses of the two varieties under non-fluctuating (NF) and fluctuating (F) light conditions with (HL) or without (LL) saturating photosynthetic photon flux (PPFD) at noon (Supplementary Figure S1; Sakowska et al., 2018). In LL, the LED system was set to simulate a fixed daily profile (June 21st in Udine, Italy; latitude: 46.07 N; longitude: 13.23 E) with a maximum PPFD at noon of 650 μmol m^−2^ s^−1^ in six chambers (three MinnGold and three Eiko; non-fluctuating light treatment—NF), whereas in the other six chambers light was fluctuated every minute ±20% around the hourly value set in NF (fluctuating light treatment—F). In HL, the light was set to reach saturating light intensity at noon with a maximum PPFD of 1,000 μmol m^−2^ s^−1^ in NF and fluctuations around the hourly value were increased up to ±50% in F. The 1-min fluctuations have been chosen to stress the photosynthesis itself (in particular the light phase) rather than other processes, such as stomatal conductance whose responses are an order of magnitude slower (Vialet-Chabrand et al., 2017b). In both experiments, all the plants received the same amount of daily PPFD (14 h per day). Transmitted light (tPPFD) was continuously measured within each chamber using a solar bar placed horizontally at the bottom of the canopy. Each bar was made of eight photodiodes in parallel (model S1087-01, Hamamatsu Photonics, Japan) with a 100 Ω resistance and was calibrated against a reference quantum sensor (Li-190R, Licor, United States) before setting up the system (Salvatori et al., 2021). Albedo (i.e., the ratio between reflected and incident radiation) was weekly measured. Then, adsorbed radiation (aPPFD) was computed as:


$$\mathrm{aPPFD}=\mathrm{PPFD}•\left(1–\mathrm{albedo}\right)–\mathrm{rPPFD}-\mathrm{tPPFD}$$

We placed eights pots per chamber in the LL treatment and 16 pots per chamber in HL. In LL only 8 pots were placed within the chambers since the used MinnGold seeds had a lower germination rate probably due to their previous storage. Measurements were run for a total of 24 days in LL and 4 weeks in the HL starting from germination. During the measurements, plants were regularly watered with the addition of a Hoagland solution twice per week (Supplementary Table S1).

At the end of the experiment, all plants within each chamber were sampled in LL, while four randomly selected plants per chamber were harvested in HL. Leaf area was measured using a LI-3000 (Licor, United States), roots were separated from stems and gently washed to remove the sands. Leaves, stems, roots, and pods were then dried at 70°C for 48 h and weighed to determine the final dry biomass which was finally scaled to the square meter.

Canopy net CO_2_ flux (A) and evapotranspiration (E) were continuously monitored (Salvatori et al., 2021): each chamber was sampled once per hour for 290 s and A was calculated as an average between 110 and 290 s, thus to not consider the tube’s purging after chamber switch (110 s). At the end of every hour, a matching procedure was applied to compute the difference in CO_2_ and H_2_O concentration between cell A and B of the LI-7000 gas analyzer and correct the measured data. The specific equations used to compute A and E are reported in the Supplementary material. As we used an inert substrate, the measured A correspond to NPP (i.e., heterotrophic respiration was cancelled). Air temperature and transmitted light across the canopy were monitored every second within each chamber using thermistors and a solar bar (Salvatori et al., 2021).

Based on flux measurements, water use efficiency (WUE) was calculated according to Bramley et al. (2013):


$$WUE=\frac{A}{E}$$

where *A* is expressed in gCO_2_ and *E* is expressed in gH_2_O. Stomatal conductance was calculated as the ratio between *E* and vapor pressure deficit (vpd; Monteith and Unsworth, 2013) calculated as:


$$vpd=es•\frac{1-rh}{P}$$

where *es* is the saturation vapor pressure (kPa), rh is the relative humidity (%) and *P* is the air pressure (kPa). These variables were calculated according to the following equations:


$$es=0.611•\exp^{\left(17.502•\frac{\mathrm{tleaf}}{\mathrm{tleaf}+240.97}\right)}$$

$$rh=\frac{ea}{es}=\frac{\frac{H_{2}O_{\mathrm{chamber}}}{1000}}{es}•P$$

where *H*_2_*O*_chamber_ is the instantaneous water concentration within the chamber (mmol H_2_O m^−3^). In all these calculations, leaf temperature ( tleaf ) was assumed to be equal to the air temperature within the chamber even though we are aware that this assumption might not be completely fulfilled when considering varieties with different albedo.

### Induction Curves at Canopy Level

In HL, before the end of the experiment, we performed a two-day light induction experiment using the plants grown in NF to estimate the time needed by the canopy to adjust to light changes and therefore reach steady-state C assimilation. Differently from the imposed growing conditions, we simulated what is usually measured at leaf level when performing a light induction curve. Plants were dark-adapted overnight and then the light was turned on and kept constant at 1000 μmol m^−2^ s^−1^ for 90 min. The induction curves were determined by fitting the following curve on the measured data:


$$A=a+\frac{b}{1+\exp\left(-\left(\frac{t-c}{d}\right)\right)}$$

where *A* is net C assimilation, *t* is time (seconds), *a*, *b* and *c* are fitting parameters. The time needed to reach a steady state was calculated as:


$$\tau =c-d•\ln\left(\frac{b}{0.95•\left(b+a\right)-a}\right)-1$$

All the fittings were done in MATLAB2019 (fminsearch).

### Gas Exchange and Fluorescence Measurements at the Leaf Level

Before final harvest, we randomly selected three plants grown in NF for either LL or HL. Three fully expanded leaves were selected to measure leaf gas exchanges using a LI-6400 (Licor Biosciences, Nebraska, United States) equipped with infrared gas analyzers (IRGA) coupled with pulse-amplitude modulation (PAM) fluorometer. Before the measurements, all plants were dark-adapted overnight. We first measured the light response curves (A/PPFD) for both varieties by measuring A at different decreasing PPFD levels (3,000; 2,500; 2,000; 1,500; 1,200; 900; 600; 300; 150; 100; 50; and 0 μmol m^−2^ s^−1^). During the measurements, CO_2_ concentration within the cuvette was maintained constant at 400 ppm, vpd at 1.8 kPa, and leaf temperature at 25°C. Then, we did a second experiment aimed at quantifying the light induction curves at leaf level under fluctuating light. Plants were dark-adapted overnight and then a fluctuating light was applied to the leaf for 60 min: 520–780 μmol m^−2^ s^−1^ with a period of 60 s in LL; 500–1,500 μmol m^−2^ s^−1^ with a period of 60 s in HL. Again, CO_2_ concentration within the cuvette was maintained constant at 400 ppm, vpd at 1.8 kPa, and leaf temperature at 25°C. Throughout the protocol a saturating light of >6,000 μmol m^−2^ s^−1^ was pulsed on to the leaf sample for 800 ms every 20 s, enabling the quantification of maximal fluorescence in the light ( $F_{m}^{\prime }$ ) and dark ( $F_{m}$ ). The maximal efficiency of PSII (F_v_/F_m_) was calculated according to (Kitajima and Butler, 1975)


$$F_{v}/F_{m}=\frac{\left(F_{m}-F_{0}\right)}{F_{m}}$$

The operating efficiency of the photosystem 2 ( $\Phi_{\mathrm{PSII}}$ ) was calculated using (Genty et al., 1989)


$$\Phi_{\mathrm{PSII}}=\frac{F_{m}^{\prime }-F_{s}}{F_{m}^{\prime }}$$

where $F_{s}$ is the steady-state F_yield_ recorded after the measuring beam under actinic light. The non-photochemical quenching (NPQ) was instead calculated using the following equation:


$$NPQ=\frac{F_{m}-F_{m}^{\prime }}{F_{m}^{\prime }}$$

adopted from (Bilger and Björkman, 1990) based on the Stern–Volmer method.

Finally, the electron transport rates estimated by fluorescence (ETR) were calculated as follows (Krall and Edwards, 1992)


$$ETR=I•\alpha •\mathrm{fractio}n_{\mathrm{PSII}}•\Phi_{\mathrm{PSII}}$$

ETR is a function of the incident light ( I ), the fraction of absorbed light received by the photosystem 2 ( $\mathrm{fractio}n_{\mathrm{PSII}}$ ) which is normally set to 0.5 (Baker, 2008) and the absorbance coefficient (α) which was set to 0.55 for MinnGold and 0.78 for Eiko as calculated in the growth chambers (Salvatori et al., 2021).

The same equations used for the canopy level data have been used to fit the data and calculate the time to reach steady state ( τ ) both in the light induction and in the high light–low light transitions (relaxation time).

### Analysis of the Data and Statistical Analysis

All the raw data have been pre-processed and analyzed with Stata10 to calculate final fluxes and derived variables according to the methodology described in Salvatori et al. (2021). The statistical analyses (*t*-test or one-way ANOVA with Duncan test) and all the graphs have been done using R.

## Results

DYNAMISM allowed continuous flux measurements under quite constant and homogenous environmental conditions (temperature and humidity) among the applied treatments (Supplementary Figure S2).

**Figure 1:**
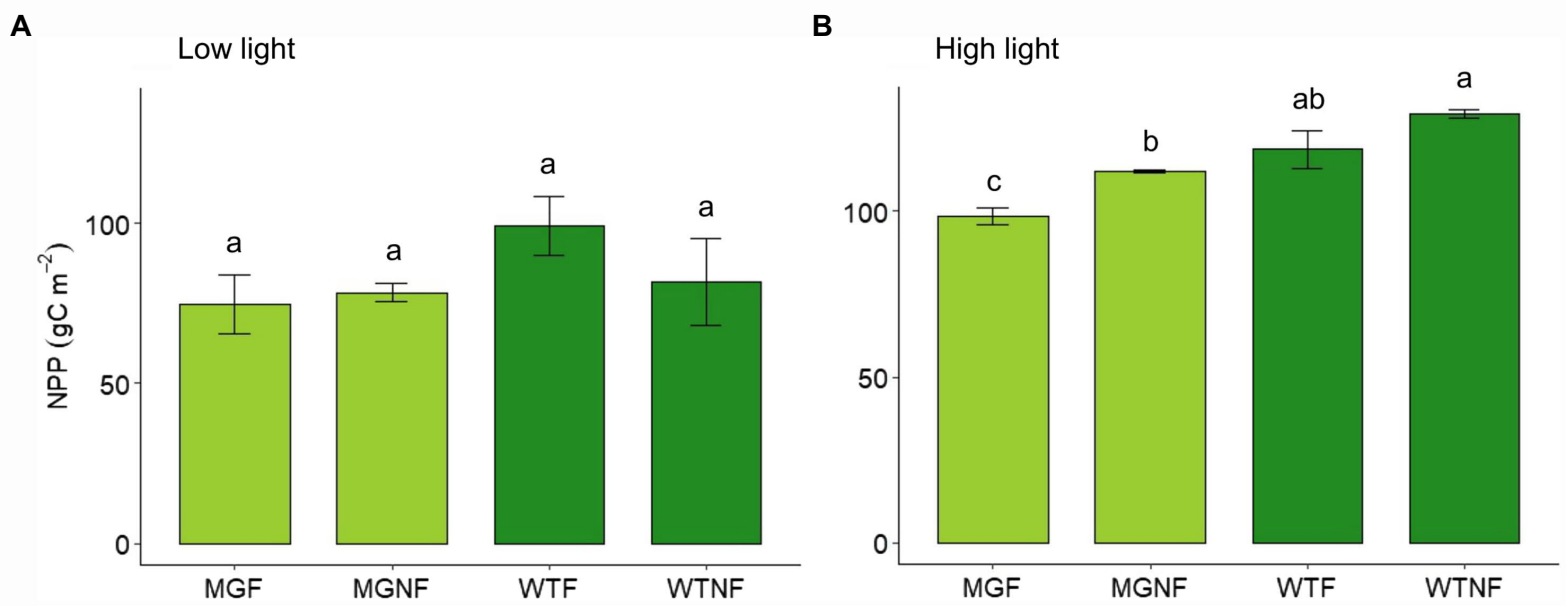
Total net primary production (NPP) for MinnGold and Wildtype (Eiko) in non-fluctuating and fluctuating light conditions in LL **(A)** and HL **(B)**. MGNF, MinnGold in non-fluctuating light; MGF, MinnGold in fluctuating light; WTNF, Wildtype (Eiko) in non-fluctuating light; WTF, Wildtype (Eiko) in fluctuating light. Different letters indicate a significant difference (one-way ANOVA followed by Duncan test). Vertical bars are standard error (*n* = 3).

In LL, no statistical difference in total NPP was found among F and NF light conditions, as well as between the two varieties, 24 days after germination (*p* > 0.05; Figure 1A). On the contrary, a significant difference was found under NF light conditions in HL (Figure 1B; Supplementary Table S2). As well, a reduction in cumulative NPP under fluctuating light (F) was evident in both the varieties even though it was significant in MinnGold only (Figure 1B; Supplementary Table S2).

**Figure 2:**
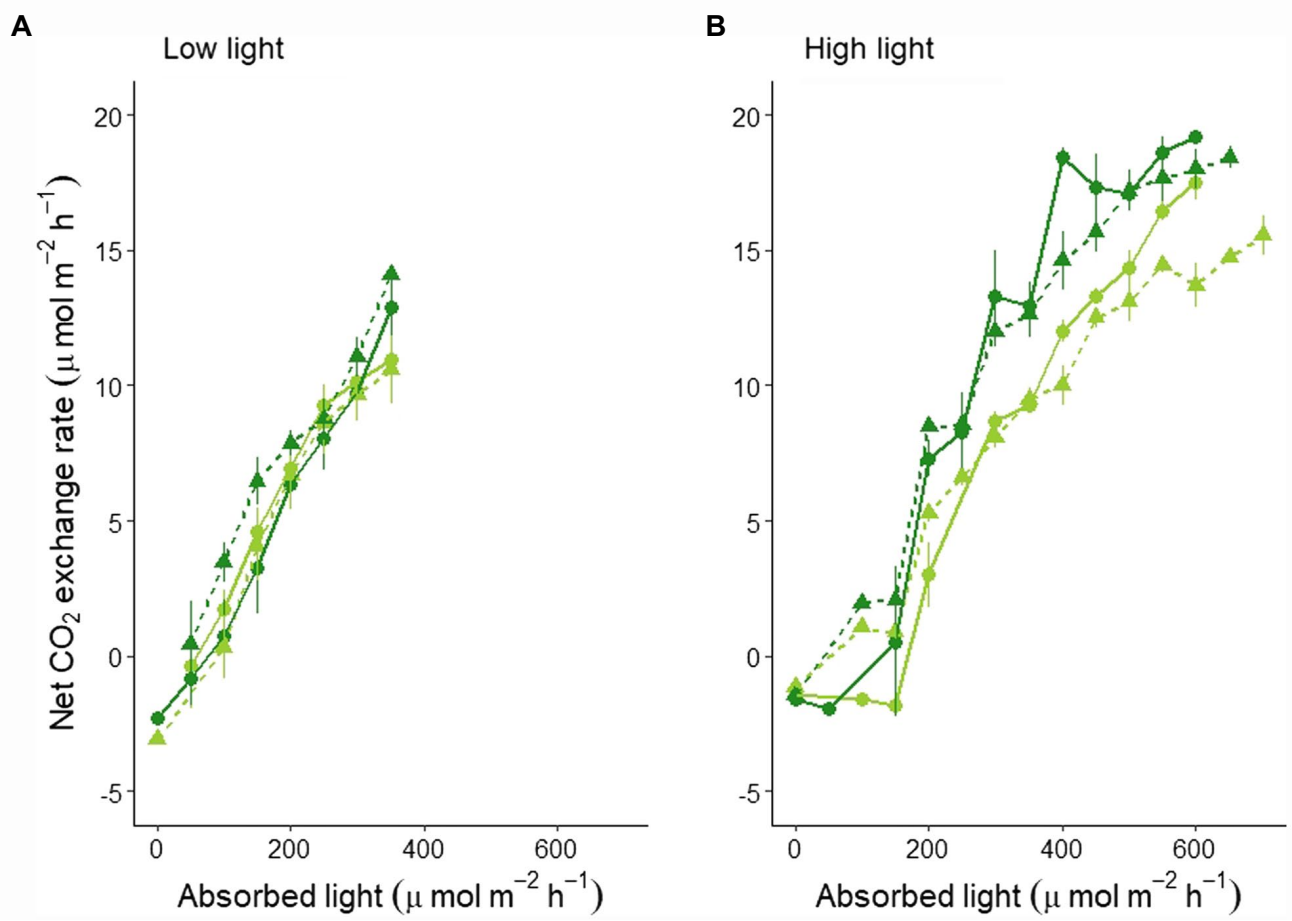
Canopy light curves based on absorbed light as an average of 4 days with similar absorbed light in LL **(A)** and HL **(B)**. The absorbed light values are averaged in classes of 50 μmol m^−2^ s^−1^ (horizontal error bars not shown for graphical reasons). The full line indicates the mean, the vertical bars indicate the standard error. The lines represent both MinnGold (light green) and Eiko (dark green) in non-fluctuating (continuous line, circular dots) and fluctuating (dashed line, triangular dots) light.

These differences were even more clear when looking at the canopy’s light curves: No significant difference between the two varieties and light regimes was found for the LL experiment (Figure 2A; statistics in Supplementary Table S3), while a lower light curve was determined for MinnGold with a significant reduction in CO_2_ under F light conditions in HL experiment (Figure 2B; statistics in Supplementary Table S3).

**Figure 3:**
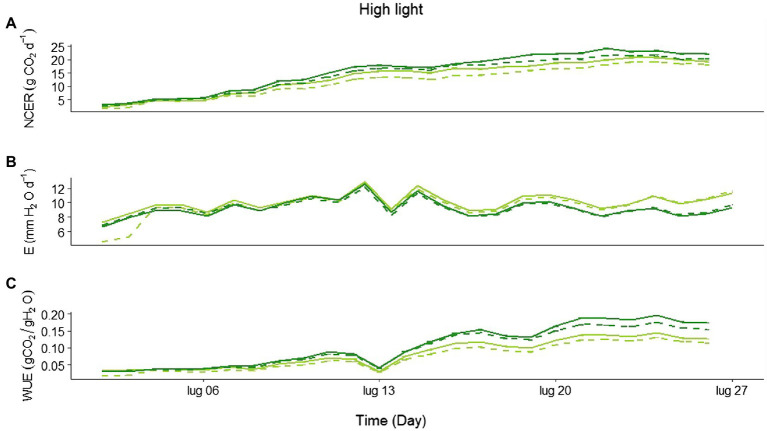
**(A)** Average daily net CO_2_ exchange rate (NECR; gCO_2_ m^−2^ d^−1^), **(B)** evapotranspiration (E; mm H_2_O d^−1^), and **(C)** water use efficiency (WUE) for all the experimental period in high light experiment (HL) for MinnGold (light green) and Eiko (dark green) in non-fluctuating (continuous line) and fluctuating (dashed line) light.

Looking in more detail at the daily CO_2_ assimilation during the HL experiment, the treatments started diverging, with a higher net C uptake in Eiko than in MinnGold, and in the NF compared to the F (Figure 3A; statistics in Supplementary Table S4), 2 weeks after emergence. Similarly, a divergence in E was also observed, with MinnGold having higher transpiration rates than Eiko (Figure 3B; statistics in Supplementary Table S4). This led to a higher WUE (Figure 3C; statistics in Supplementary Table S4) for Eiko in NF when compared to all the other treatments.

**Figure 4:**
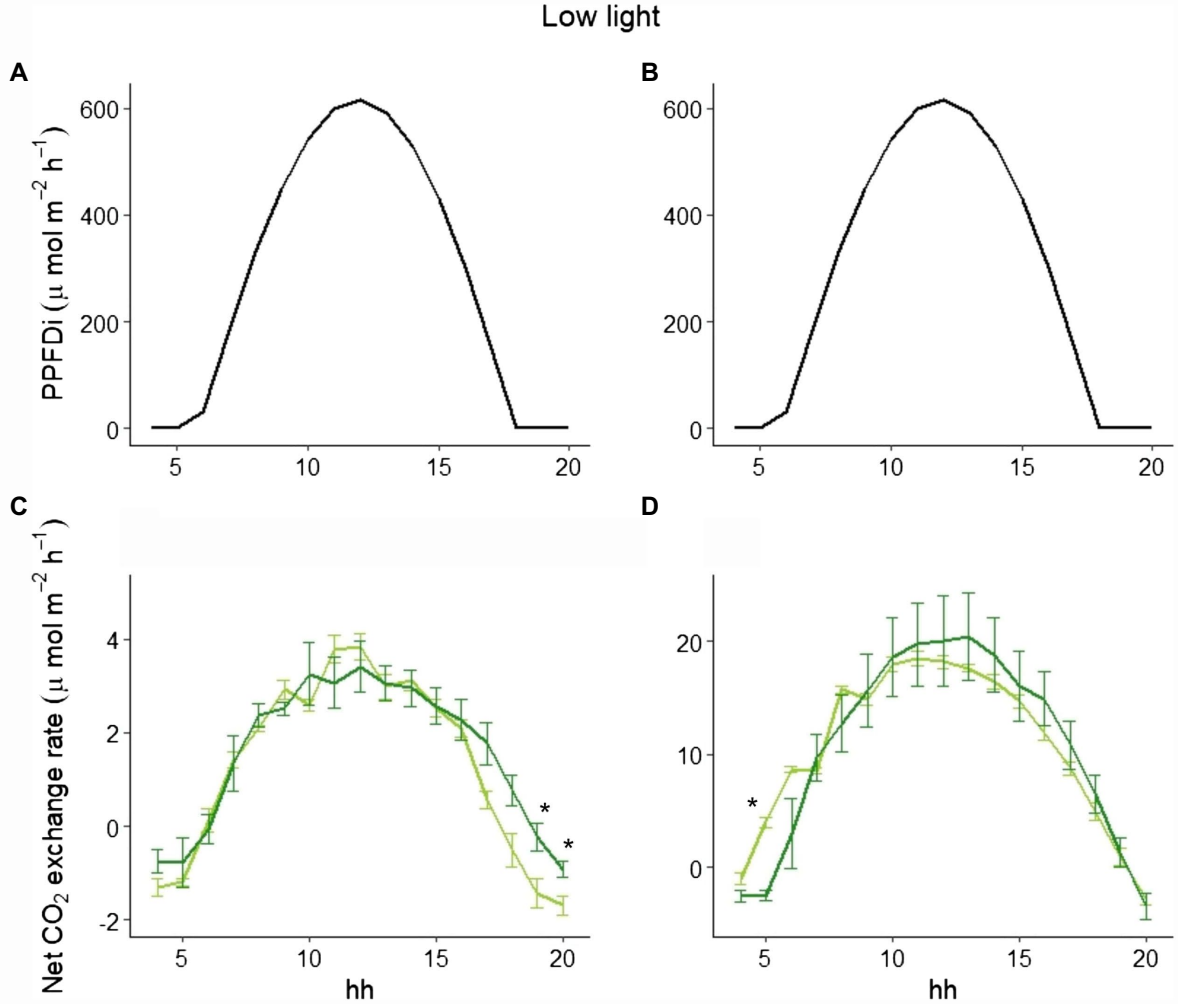
Incident PPFD and hourly net CO_2_ exchange for MinnGold (light green) and Eiko (dark green) under non-fluctuating light in the LL experiment, 9 (**A**,**C**) and 28 (**B**,**D**) days after germination. Asterisks indicate a statistical difference between the two varieties determined with a *t*-test. Vertical bars indicate standard errors (*n* = 3).

**Figure 5:**
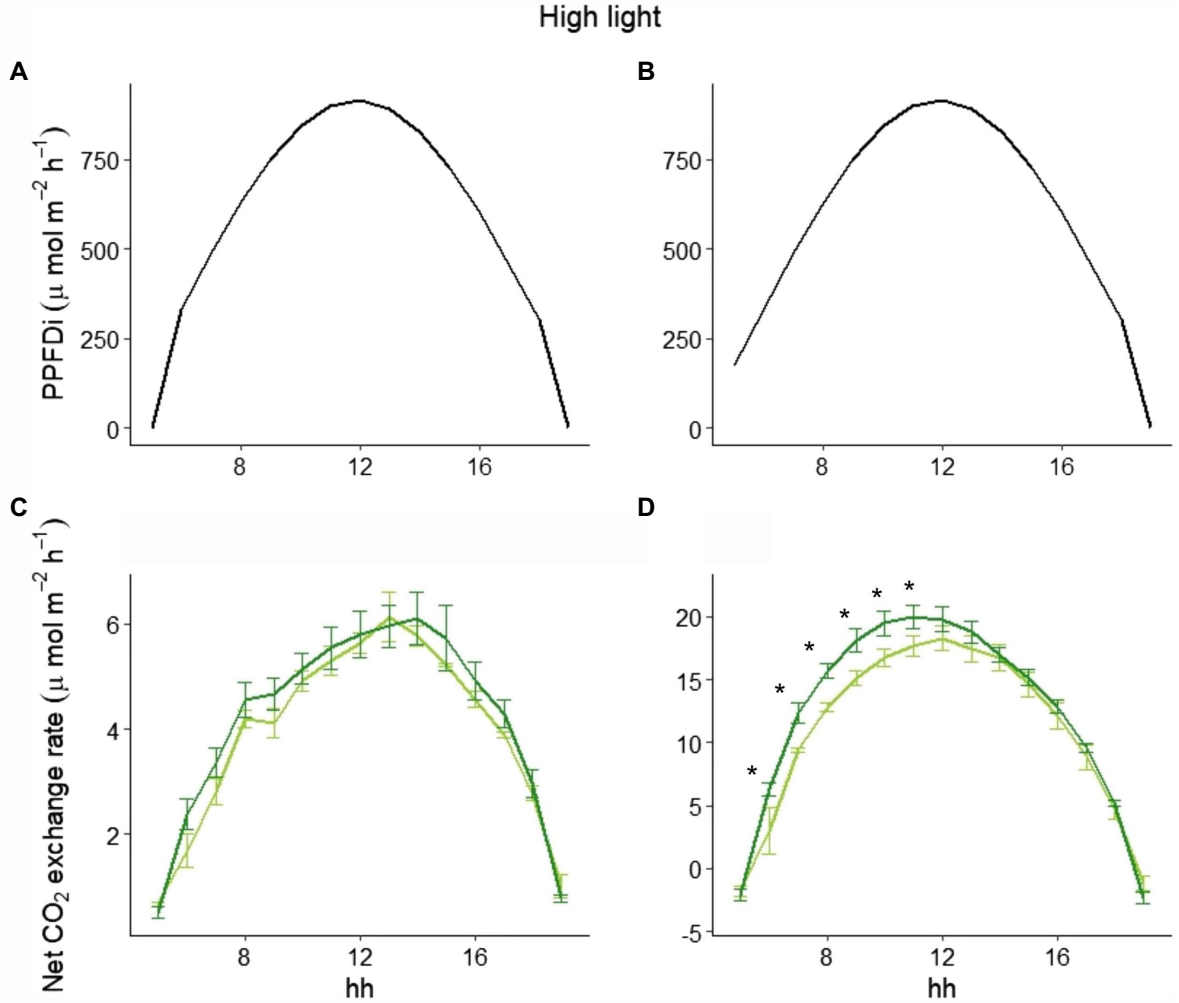
Incident PPFD and hourly net CO_2_ exchange for MinnGold (light green) and Eiko (dark green) under non-fluctuating light in the HL experiment, 9 (**A**,**C**) and 28 (**B**,**D**) days after germination. Asterisks indicate a statistical difference between the two varieties determined with a *t*-test. Vertical bars indicate standard errors (*n* = 3).

To explain the different behavior of total NPP between LL and HL (Figure 1), we selected three representative days in the beginning, in the middle, and at the end of the experimental period in both LL and HL. Looking at those data, while the hourly net CO_2_ exchange was similar between the two varieties in LL either at the beginning or the end of the experiment (Figure 4), a clear difference between the varieties in the two periods was evident in HL. In fact, at the beginning of the experiment (i.e., 9 days after germination), no difference occurred during the day (Figures 5A,C), whereas a significant difference was evident during the morning starting from the 16th day after germination. Such a difference was exacerbated in the last day of the experiment (i.e., 28 days after germination; Figures 5B,D): a clear hysteresis was observed in MinnGold with higher C uptake in the afternoon than in the morning given the same amount of light (Supplementary Figure S3).

The difference in C assimilation during the morning could be due to a different adjustment of the leaves inside the canopy after the light is turned on, with an impact on absorbed radiation and then on photosynthesis. However, the velocity by which the two varieties reached minimum light transmittance 1 h after the light was turned on was not significantly different (Supplementary Figure S4).

We then investigated the possible impact of photosynthetic induction. In fact, even though the two varieties showed similar light curves (Supplementary Figure S1), we could not exclude that they behave differently in dark–light transitions. Therefore, we performed an induction experiment at both leaf and canopy scales to estimate the time to reach steady-state C assimilation (*τ*). No significant difference between MinnGold and Eiko was found either in terms of velocity of induction or in terms of steady-state values of C assimilation at both leaf and canopy scales (Table 1).

**Table 1:** Results of the induction experiment on MinnGold and Eiko plants grown in HL without light fluctuations (NF) at leaf (n = 4) and canopy (n = 3) scale.

	Leaf			Canopy		
	MinnGold	Eiko	value of *p*	MinnGold	Eiko	value of *p*
*A_ss_*	15.1 ± 1.5	13.4 ± 2.2	0.20	15.4 ± 2.7	17.4 ± 3.5	0.48
*τ*	32.1 ± 5.1	37.9 ± 6.7	0.52	18.2 ± 1.8	21.2 ± 3.3	0.48

The values of net CO_2_ assimilation at steady state (A_ss_ in μmol CO_2_ m^−2^ s^−1^) and the time to reach steady state (τ in minutes) are reported. The value of *p* is the result of a *t*-test. Mean ± standard error.

## Discussion

The effect of dynamic photosynthesis on the overall carbon (C) uptake is still a matter of discussion. Photosynthesis under fluctuating light is in a “roller coaster ride” (Kaiser et al., 2018b), which is generally thought to reduce the daily integral C assimilation. For this reason, several efforts have been taken to find suitable methods to accelerate the most limiting process in photosynthesis (Armbruster et al., 2014; Kromdijk et al., 2016; Kaiser et al., 2019; Papanatsiou et al., 2019), but still with contrasting results (Kromdijk et al., 2016; Garcia-Molina and Leister, 2020). In the present paper, we investigated the effect of fluctuating light in both low (LL) and high light (HL) for a soybean wildtype and a chlorophyll deficient soybean variety.

Under non-fluctuating light conditions (NF), the two cultivars showed no differences in total C uptake (Figure 1A), as well as in their canopy light curves, in LL (Figure 2A). On the contrary, a significant difference was found in HL (Figures 1B, 2B): the two varieties started diverging 2 weeks after germination and instantaneous fluxes showed a significant difference early in the morning only (Figure 5D). These contradictory results were unexpected since the two varieties showed similar light curves (Supplementary Figure S1), A/Ci curves (Sakowska et al., 2018), and photosynthetic induction curves (Table 1) at both leaf and canopy scale. These measurements at both LL and HL would exclude a different velocity in Rubisco activation, which is usually considered limiting in the light-saturated phase (Murchie and Ruban, 2019). Nevertheless, it is worth mentioning that in certain conditions MinnGold is found to have a slower induction curve than Eiko (Salvatori et al., 2022), and therefore this needs to be further investigated. Our further measurements related to light transmission through the canopy (Supplementary Figure S4) showed that the changes in transmitted light are faster in Eiko than in MinnGold (but not significantly) and therefore the detected difference in C assimilation during the morning could have been due, to a small extent, to a different adjustment of the leaves inside the canopy as suggested by (Kaiser et al., 2014; Morales et al., 2018). Furthermore, the measured reduction in total C assimilation in MinnGold in HL, but not in LL, might be related to a higher photo-inhibition. In fact, Ferroni et al. (2020) have reported that changes in photosystem stoichiometry in chlorophyll deficient wheats might be a reason for a reduced photoprotection. In this regard, Minngold is known to have a large truncation in the PSII antennae compared to PSI leading to higher chl a/b and PSII/PSI ratios (Slattery et al., 2017). This impairment can disturb the assembly of the light-harvesting (LH) component (Falbel et al., 1996) and entail an altered electron transport (Ferroni et al., 2020). In the Chl-deficient mutant, we found a significantly lower non-photochemical quenching (NPQ) in both LL and HL (Supplementary Figure S5). This low NPQ would allow MinnGold to have the same quantum yield (and related C assimilation) as Eiko in non-saturating light intensities (LL), but it might not be sufficient to protect plants from photo-inhibition in saturating light conditions (HL). In other words, this means that, in absence of any photo-inhibition, the two varieties have a similar control on the light phase of photosynthesis (Murchie and Ruban, 2019). Nevertheless, when calculating the maximal efficiency of PSII (F_v_/F_m_), a reduction was found for MinnGold (in particular in HL), even if not significant (Supplementary Table S5). As previous studies have shown that enhancing the capacity for NPQ resulted in increased yield and biomass in rice (Hubbart et al., 2018), a similar approach aimed at increasing NPQ in MinnGold might eventually reduce the negative effects of photo-inhibition at the whole plant level, also in HL. Nevertheless, we cannot exclude that this observed difference is only due to a lower absorbed light causing a non-detectable difference in LL (Figure 1A), which becomes evident only in HL conditions (Figure 1B). Furthermore, all these results call for the hypothesis that several processes are limiting C assimilation in MinnGold, and an unambiguous mechanism cannot be identified.

It is in general thought that light fluctuations reduce the overall C gain because of the lagging response of the photosynthetic machinery (Kaiser et al., 2018a; Slattery et al., 2018). Kromdijk et al. (2016) have also shown that the bioengineered acceleration of the recovery from photo-inhibition increases the overall C uptake under fluctuating light in tobacco plants. On the contrary to our second working hypothesis, our experimental data support the idea that dynamic photosynthesis is not really detrimental in green varieties continuously grown under fluctuating light (Vialet-Chabrand et al., 2017a; Marler, 2020), either in LL or HL (Figure 1B), because of possible plants’ dynamic and developmental acclimations to ensure optimum resource use changes in their environment (Gjindali et al., 2021). Li et al. (2021) have also shown that photosynthetic different acclimation strategies in wheat can maximizes C assimilation under intermittent high light conditions, and this might also be the case for soybean.

A different pattern in MinnGold was found when compared to Eiko. In fact, while we found no effect of light fluctuations on biomass accumulation in LL (Figure 1A), we measured a further decrease in cumulative C uptake for MinnGold in HL (Figure 1B). Leaf level data and models (Salvatori et al., 2022) taken in fluctuating LL confirmed that no difference is expected in such conditions, while the measured reduction in C uptake in MinnGold in HL might be due to several processes, such as lack of acclimation; chloroplasts movements within the leaves (Kaiser et al., 2014; Dutta et al., 2015; Semer et al., 2018); photoprotective mechanisms to prevent photo-inhibition (Eberhard et al., 2008; Yamori, 2016); leaf movement within the canopy (Gamon and Pearcy, 1989; Huang et al., 2014; Kaiser et al., 2014); different NPQ relaxation upon transition to low light (Zhu et al., 2004; Sakowska et al., 2018); and lower NPQ in the low light–high light transitions causing photo-inhibition (Yamori, 2016). Even though our experimental setup was not able to clearly elucidate the role of all these possible mechanisms, combining our results under LL and HL (i.e., photosynthetic rates, leaf movements, induction curves) with previous findings (Sakowska et al., 2018; Ferroni et al., 2020) suggests that the truncation in the PSII would impair the global reprogramming of photosynthetic gene expression. Therefore, even if MinnGold endures smaller abrupt changes in light intensity, the truncation in PSII might induce a less efficient electron transport and slower NPQ induction/relaxation dynamics which might have an important role on photo-inhibition and C assimilation in MinnGold.

## Conclusion

Contrarily to our initial hypothesis, dynamic photosynthesis seems to be detrimental to overall C uptake at canopy scale only for the chlorophyll deficient variety (MinnGold) in high light conditions, while the green variety (Eiko) seems to be well-adapted to light fluctuations in both low and high light. The fact that fluctuating light has been often reported to decrease C uptake in plants might be due to artifacts in the adopted protocols, which were not able to properly isolate the effects of dynamic light (Marler, 2020). Our experiment did consider these last only and support the idea of new and more promising opportunities for the selection of greater crop photosynthetic efficiency by focusing on the variation in non-steady-state efficiency (Acevedo-Siaca et al., 2020).

These preliminary findings call for further experiments at the canopy level involving different green species and, possibly, their chlorophyll deficient isolines. Further experiments investigating other varieties and fluctuations regimes are necessary to unravel the role of the chlorophyll content in the adaptation to light fluctuation. Furthermore, to allow a better comparison between varieties and to improve photosynthesis in such crops under natural conditions, it would be necessary to obtain data relative to their antenna structure. Finally, identifying varieties already well adapted to fluctuations of light serves both to target those phenotypes with improved growth rate in field conditions, and to target the genes responsible for the global reprogramming of the photosynthetic machinery in fluctuating light environments is needed.

## Data Availability Statement

The raw data supporting the conclusions of this article will be made available by the authors, without undue reservation.

## Author Contributions

NS performed the experiment and wrote the manuscript. GA and NS collected the data and performed the data analysis. OM provided the facilities for the experiment. GA, OM, and AP designed the research. All the authors contributed to data interpretation and to the final version of the manuscript.

## Funding

GA was supported by funds of the University of Udine for his mission to Forschungszentrum Jülich GmbH for the system development (PDM_VQR3_DI4A_MISSIONI).

## Conflict of Interest

The authors declare that the research was conducted in the absence of any commercial or financial relationships that could be construed as a potential conflict of interest.

## Publisher’s Note

All claims expressed in this article are solely those of the authors and do not necessarily represent those of their affiliated organizations, or those of the publisher, the editors and the reviewers. Any product that may be evaluated in this article, or claim that may be made by its manufacturer, is not guaranteed or endorsed by the publisher.

## References

[ref600] Armbruster U Carrillo L. R. Venema K. Pavlovic L. Schmidtmann E. Kornfeld A.. (2014). Ion antiport accelerates photosynthetic acclimation in fluctuating light environments. Nat. Commun. 5:5439. doi: 10.1038/ncomms6439, PMID: 25451040 PMC4243252

[ref1] Acevedo-Siaca L. G. Coe R. Wang Y. Kromdijk J. Quick W. P. Long S. P. (2020). Variation in photosynthetic induction between rice accessions and its potential for improving productivity. New Phytol. 227, 1097–1108. doi: 10.1111/nph.16454, PMID: 32124982 PMC7383871

[ref2] Alter P. Dreissen A. Luo F. L. Matsubara S. (2012). Acclimatory responses of Arabidopsis to fluctuating light environment: comparison of different sunfleck regimes and accessions. Photosynth. Res. 113, 221–237. doi: 10.1007/s11120-012-9757-2, PMID: 22729524 PMC3430843

[ref3] Baker N. R. (2008). Chlorophyll fluorescence: A probe of photosynthesis In vivo. Annu. Rev. Plant Biol. 59, 89–113. doi: 10.1146/annurev.arplant.59.032607.092759, PMID: 18444897

[ref4] Bilger W. Björkman O. (1990). Role of the xanthophyll cycle in photoprotection elucidated by measurements of light-induced absorbance changes, fluorescence and photosynthesis in leaves of Hedera canariensis. Photosynth. Res. 25, 173–185. doi: 10.1007/BF00033159, PMID: 24420348

[ref5] Bramley H. Turner N. C. Siddique K. H. M. (2013). “Water use efficiency,” in Genomics and Breeding for Climate-Resilient Crops. ed. Kole C. (Berlin, Heidelberg: Springer), 225–268.

[ref6] Campbell B. W. Mani D. Curtin S. J. Slattery R. A. Michno J. M. Ort D. R.. (2015). Identical substitutions in magnesium chelatase paralogs result in chlorophyll-deficient soybean mutants. G3 5, 123–131. doi: 10.1534/g3.114.015255, PMID: 25452420 PMC4291463

[ref7] Dutta S. Cruz J. A. Jiao Y. Chen J. Kramer D. M. Osteryoung K. W. (2015). Non-invasive, whole-plant imaging of chloroplast movement and chlorophyll fluorescence reveals photosynthetic phenotypes independent of chloroplast photorelocation defects in chloroplast division mutants. Plant J. 84, 428–442. doi: 10.1111/tpj.13009, PMID: 26332826

[ref8] Eberhard S. Finazzi G. Wollman F.-A. (2008). The dynamics of photosynthesis. Annu. Rev. Genet. 42, 463–515. doi: 10.1146/annurev.genet.42.110807.091452 18983262

[ref9] Falbel T. G. Meehl J. B. Staehelin L. A. (1996). Severity of mutant phenotype in a series of chlorophyll-deficient wheat mutants depends on light intensity and the severity of the block in chlorophyll synthesis. Plant Physiol. 112, 821–832. doi: 10.1104/pp.112.2.821, PMID: 8883392 PMC158007

[ref10] Ferroni L. Živčak M. Sytar O. Kovár M. Watanabe N. Pancaldi S.. (2020). Chlorophyll-depleted wheat mutants are disturbed in photosynthetic electron flow regulation but can retain an acclimation ability to a fluctuating light regime. Environ. Exp. Bot. 178:104156. doi: 10.1016/j.envexpbot.2020.104156

[ref11] Flexas J. Loreto F. Medrano H. (eds.) (2012). Terrestrial photosynthesis in a changing environment: a molecular, physiological, and ecological approach. Cambridge University Press.

[ref12] Foyer C. H. Ruban A. V. Nixon P. J. (2017). Photosynthesis solutions to enhance productivity. Philos. Trans. R Soc. B Biol. Sci. 372:20160374. doi: 10.1098/rstb.2016.0374, PMID: 28808094 PMC5566875

[ref13] Gamon J. A. Pearcy R. W. (1989). Leaf movement, stress avoidance and photosynthesis in Vitis californica. Oecologia 79, 475–481. doi: 10.1007/BF00378664, PMID: 28313481

[ref14] Garcia-Molina A. Leister D. (2020). Accelerated relaxation of photoprotection impairs biomass accumulation in Arabidopsis. Nat. Plants 6, 9–12. doi: 10.1038/s41477-019-0572-z, PMID: 31907400

[ref15] Genty B. Briantais J. M. Baker N. R. (1989). The relationship between the quantum yield of photosynthetic electron transport and quenching of chlorophyll fluorescence. Biochim. Biophys. Acta Gen. Subj. 990, 87–92. doi: 10.1016/S0304-4165(89)80016-9

[ref16] Gjindali A. Herrmann H. A. Schwartz J.-M. Johnson G. N. Calzadilla P. I. (2021). A holistic approach to study photosynthetic acclimation responses of plants to fluctuating light. Front. Plant Sci. 12:668512. doi: 10.3389/fpls.2021.668512, PMID: 33936157 PMC8079764

[ref17] Graham P. J. Nguyen B. Burdyny T. Sinton D. (2017). A penalty on photosynthetic growth in fluctuating light. Sci. Rep. 7, 12513–12511. doi: 10.1038/s41598-017-12923-1, PMID: 28970553 PMC5624943

[ref18] Horton P. Ruban A. V. Walters R. G. (1996). Regulation of light harvesting in green plants. Annu. Rev. Plant Physiol. Plant Mol. Biol. 47, 655–684. doi: 10.1146/annurev.arplant.47.1.655 15012304

[ref19] Huang W. Zhang J. L. Zhang S. B. Hu H. (2014). Evidence for the regulation of leaf movement by photosystem II activity. Environ. Exp. Bot. 107, 167–172. doi: 10.1016/j.envexpbot.2014.06.010

[ref20] Hubbart S. Smillie I. R. A. Heatley M. Swarup R. Foo C. C. Zhao L.. (2018). Enhanced thylakoid photoprotection can increase yield and canopy radiation use efficiency in rice. Commun. Biol. 1, 22–12. doi: 10.1038/s42003-018-0026-6, PMID: 30271909 PMC6123638

[ref21] Kaiser E. Galvis V. C. Armbruster U. (2019). Efficient photosynthesis in dynamic light environments: A chloroplast’s perspective. Biochem. J. 476, 2725–2741. doi: 10.1042/BCJ20190134, PMID: 31654058 PMC6792033

[ref22] Kaiser E. Matsubara S. Harbinson J. Heuvelink E. Marcelis L. F. M. (2018a). Acclimation of photosynthesis to lightflecks in tomato leaves: interaction with progressive shading in a growing canopy. Physiol. Plant. 162, 506–517. doi: 10.1111/ppl.12668, PMID: 29125181

[ref23] Kaiser E. Morales A. Harbinson J. (2018b). Fluctuating light takes crop photosynthesis on a rollercoaster ride. Plant Physiol. 176, 977–989. doi: 10.1104/pp.17.01250, PMID: 29046421 PMC5813579

[ref24] Kaiser E. Morales A. Harbinson J. Heuvelink E. Kromdijk J. Marcelis L. F. M. (2014). Dynamic photosynthesis in different environmental conditions. J. Exp. Bot. 66, 2415–2426. doi: 10.1093/jxb/eru406 25324402

[ref25] Kaiser E. Walther D. Armbruster U. (2020). Growth under fluctuating light reveals large trait variation in a panel of *Arabidopsis* accessions. Plan. Theor. 9:319. doi: 10.3390/plants9030319, PMID: 32138234 PMC7154909

[ref26] Kitajima M. B. W. L. Butler W. L. (1975). Quenching of chlorophyll fluorescence and primary photochemistry in chloroplasts by dibromothymoquinone. BBA Bioenerg. 376, 105–115. doi: 10.1016/0005-2728(75)90209-1, PMID: 1125215

[ref27] Krall J. P. Edwards G. E. (1992). Relationship between photosystem II activity and CO2 fixation in leaves. Physiol. Plant. 86, 180–187. doi: 10.1111/j.1399-3054.1992.tb01328.x

[ref28] Kromdijk J. Glowacka K. Leonelli L. Gabilly S. T. Iwai M. Niyogi K. K.. (2016). Improving photosynthesis and crop productivity by accelerating recovery from photoprotection. Science 354, 857–861. doi: 10.1126/science.aai8878, PMID: 27856901

[ref29] Li Y. T. Yang C. Zhang Z. S. Zhao S. J. Gao H. Y. (2021). Photosynthetic acclimation strategies in response to intermittent exposure to high light intensity in wheat (*Triticum aestivum* L.). Environ. Exp. Bot. 181:104275. doi: 10.1016/j.envexpbot.2020.104275

[ref30] Marler T. E. (2020). Artifleck: The study of artifactual responses to light flecks with inappropriate leaves. Plan. Theory 9, 1–14. doi: 10.3390/plants9070905, PMID: 32708982 PMC7412511

[ref31] Matsubara S. (2018). Growing plants in fluctuating environments: why bother? J. Exp. Bot. 69, 4651–4654. doi: 10.1093/jxb/ery312, PMID: 30307518 PMC6137991

[ref32] Matthews J. S. A. Vialet-Chabrand S. Lawson T. (2018). Acclimation to fluctuating light impacts the rapidity of response and diurnal rhythm of stomatal conductance. Plant Physiol. 176, 1939–1951. doi: 10.1104/pp.17.01809, PMID: 29371250 PMC5841698

[ref33] Mohammed G. H. Colombo R. Middleton E. M. Rascher U. van der Tol C. Nedbal L.. (2019). Remote sensing of solar-induced chlorophyll fluorescence (SIF) in vegetation: 50 years of progress. Remote Sens. Environ. 231:111177. doi: 10.1016/j.rse.2019.04.030, PMID: 33414568 PMC7787158

[ref34] Monteith J. L. Unsworth M. H. (2013). Principles of Environmental Physics. Plants, Animals and Physics. United States: Academic Press.

[ref35] Morales A. Kaiser E. (2020). Photosynthetic acclimation to fluctuating irradiance in plants. Front. Plant Sci. 11:SS. doi: 10.3389/fpls.2020.00268, PMID: 32265952 PMC7105707

[ref36] Morales A. Kaiser E. Yin X. Harbinson J. Molenaar J. Driever S. M.. (2018). Dynamic modelling of limitations on improving leaf CO2 assimilation under fluctuating irradiance. Plant Cell Environ. 41, 589–604. doi: 10.1111/pce.13119, PMID: 29243271

[ref37] Murchie E. H. Ruban A. V. (2019). Dynamic non-photochemical quenching in plants: from molecular mechanism to productivity. Plant J. 101, 885–896. doi: 10.1111/tpj.14601, PMID: 31686424

[ref38] Papanatsiou M. Petersen J. Henderson L. Wang Y. Christie J. M. Blatt M. R. (2019). Optogenetic manipulation of stomatal kinetics improves carbon assimilation, water use, and growth. Science 363, 1456–1459. doi: 10.1126/science.aaw0046, PMID: 30923223

[ref39] Pearcy R. W. (1990). Sunflecks and photosynthesis in plant canopies. Annu. Rev. Plant Biol. 41, 421–453. doi: 10.1016/0016-0032(53)91189-2

[ref40] Rascher U. Nedbal L. (2006). Dynamics of photosynthesis in fluctuating light. Curr. Opin. Plant Biol. 9, 671–678. doi: 10.1016/j.pbi.2006.09.012 17011815

[ref41] Roy J. Rineau F. De Boeck H. J. Nijs I. Pütz T. Abiven S.. (2021). Ecotrons: powerful and versatile ecosystem analysers for ecology, agronomy and environmental science. Glob. Chang. Biol. 27, 1387–1407. doi: 10.1111/gcb.15471, PMID: 33274502 PMC7986626

[ref42] Sakowska K. Alberti G. Genesio L. Peressotti A. Delle Vedove G. Gianelle D.. (2018). Leaf and canopy photosynthesis of a chlorophyll deficient soybean mutant. Plant Cell Environ. 41, 1427–1437. doi: 10.1111/pce.13180, PMID: 29498070

[ref43] Salvatori N. Carteni F. Giannino F. Alberti G. Mazzoleni S. Peressotti A. (2022). A system dynamics approach to model photosynthesis at leaf level under fluctuating light. Front. Plant Sci. 12:787877. doi: 10.3389/fpls.2021.787877, PMID: 35154180 PMC8833254

[ref44] Salvatori N. Giorgio A. Muller O. Rascher U. Peressotti A. (2021). A low-cost automated growth chamber system for continuous measurements of gas exchange at canopy scale in dynamic conditions. Plant Methods 17:69. doi: 10.1186/s13007-021-00772-z, PMID: 34193215 PMC8243713

[ref45] Semer J. Štroch M. Špunda V. Navrátil M. (2018). Partitioning of absorbed light energy within photosystem II in barley can be affected by chloroplast movement. J. Photochem. Photobiol. B Biol. 186, 98–106. doi: 10.1016/j.jphotobiol.2018.06.019, PMID: 30025290

[ref46] Sinclair T. R. Rufty T. W. Lewis R. S. (2019). Increasing photosynthesis: unlikely solution for world food problem. Trends Plant Sci. 24, 1032–1039. doi: 10.1016/j.tplants.2019.07.008, PMID: 31488354

[ref47] Slattery R. A. VanLoocke A. Bernacchi C. J. Zhu Z. G. Ort D. R. (2017). Photosynthesis, light use efficiency, and yield of reduced-chlorophyll soybean mutants in field conditions. Front. Plant Sci. 8:549. doi: 10.3389/fpls.2017.00549, PMID: 28458677 PMC5394119

[ref48] Slattery R. A. Walker B. J. Weber A. P. M. Ort D. R. (2018). The impacts of fluctuating light on crop performance. Plant Physiol. 176, 990–1003. doi: 10.1104/pp.17.01234, PMID: 29192028 PMC5813574

[ref49] Stirbet A. Lazár D. Guo Y. Govindjee. (2019). Photosynthesis: basics, history, and modeling. Ann Bot. 126, 511–537. doi: 10.1093/aob/mcz171, PMID: 31641747 PMC7489092

[ref50] Suorsa M. Järvi S. Grieco M. Nurmi M. Pietrzykowska M. Rantala M.. (2012). PROTON GRADIENT REGULATION5 is essential for proper acclimation of Arabidopsis photosystem I to naturally and artificially fluctuating light conditions. Plant Cell 24, 2934–2948. doi: 10.1105/tpc.112.097162, PMID: 22822205 PMC3426124

[ref51] Taylor S. H. Long S. P. (2017). Slow induction of photosynthesis on shade to sun transitions in wheat may cost at least 21% of productivity. Philos. Trans. R. Soc. B. Biol. Sci. 372:20160543. doi: 10.1098/rstb.2016.0543, PMID: 28808109 PMC5566890

[ref52] Vialet-Chabrand S. R. M. Matthews J. S. A. McAusland L. Blatt M. R. Griffiths H. Lawson T. (2017b). Temporal dynamics of stomatal behavior: modeling and implications for photosynthesis and water use. Plant Physiol. 174, 603–613. doi: 10.1104/pp.17.00125, PMID: 28363993 PMC5462030

[ref53] Vialet-Chabrand S. Matthews J. S. A. Simkin A. J. Raines C. A. Lawson T. (2017a). Importance of fluctuations in light on plant photosynthetic acclimation. Plant Physiol. 173, 2163–2179. doi: 10.1104/pp.16.01767, PMID: 28184008 PMC5373038

[ref54] Wang M. Guan D. X. Han S. J. Wu J. L. (2010). Comparison of eddy covariance and chamber-based methods for measuring CO2 flux in a temperate mixed forest. Tree Physiol. 30, 149–163. doi: 10.1093/treephys/tpp098, PMID: 19955193

[ref55] Wang Z. Luo C. Sauer T. J. Helmers M. J. Xu L. Horton R. (2018). Canopy chamber measurements of carbon dioxide fluxes in corn and soybean fields. Vadose Zo. J. 17, 1–5. doi: 10.2136/vzj2018.07.0130

[ref56] Yamori W. (2016). Photosynthetic response to fluctuating environments and photoprotective strategies under abiotic stress. J. Plant Res. 129, 379–395. doi: 10.1007/s10265-016-0816-1, PMID: 27023791

[ref57] Yoon D.-K. Ishiyama K. Suganami M. Tazoe Y. Watanabe M. Imaruoka S.. (2020). Transgenic rice overproducing Rubisco exhibits increased yields with improved nitrogen-use efficiency in an experimental paddy field. Nat. Food. 1, 134–139. doi: 10.1038/s43016-020-0033-x 37127998

[ref58] Zhu X. G. X.-G. Ort D. R. Whitmarsh J. Long S. P. (2004). The slow reversibility of photosystem II thermal energy dissipation on transfer from high to low light may cause large losses in carbon gain by crop canopies: A theoretical analysis. J. Exp. Bot. 55, 1167–1175. doi: 10.1093/jxb/erh141, PMID: 15133059

