# Supplementary material for: Does Fluctuating Light Affect Crop Yield? A Focus on the Dynamic Photosynthesis of Two Soybean Varieties
Source: Front Plant Sci. 2022 Apr 25;13:862275. doi: 10.3389/fpls.2022.862275 (PMC9085482; doi:10.3389/fpls.2022.862275)
Supplement: Supplementary file 1 [file Data_Sheet_1.docx]

**Supplementary**

**Calculations of canopy net CO_2_ flux (A) and evapotranspiration (E)**

E and A are computed according to the following equations:

$E=air_{flow}\cdot\frac{H_{2}O_{chamber}-H_{2}O_{in}}{S\cdot(1000-H_{2}O_{out})}$

$A=air_{flow}\cdot\frac{C{O_{2}}_{chamber}-C{O_{2}}_{in}}{S}-C{O_{2}}_{out}\cdot E$

where $H_{2}O_{in}$ and ${{CO}_{2}}_{in}$ are the H_2_O and CO_2_ concentrations within the buffer chamber (inlet) and $H_{2}O_{chamber}$ and ${{CO}_{2}}_{chamber}$ are the concentrations in each chamber; ${air}_{flow}$ is the air flux entering the chamber (mol s^-1^) and S is the chamber area (0.36 m^2^). Air flow from the miniature air flow transmitter is converted from m s^-1^ (flow) to mol s^-1^ (air_flow_) according to the equation:

$air_{flow}=\frac{flow\cdot S_{tube}\cdot P}{R\cdot(T_{chamber}+273.15)}$

where S_tube_ is the tube area (0.10 $\cdot$ 0.10 m^2^), P is the inlet air pressure (Pa) and T_chamber_ is the air temperature inside the chamber (°C) and R is the universal constant of gases (8.3144598 m^3^ Pa K^−1^ mol^−1^).

**Tables**

***Table S1.*** *Nutrients (mL) for a 100% Hoagland solution. In our experiment, we used a half strength solution: nutrients for 30L diluted in 60L of distilled water per week. pH = 6.47.*

| Components | Stock (g/L) | mL stock/30L |
| --- | --- | --- |
| Macro-nutrients |  |  |
| 1M KNO_3_ | 101 | 150 |
| 1M Ca(NO_3_)_2_ 4H_2_O | 236 | 150 |
| Fe-EDTA | 15 | 30 |
| 2M MgSO_4_ 7H_2_O | 123 | 120 |
| 1 M KH_2_PO_4_ | 136 | 30 |
| Micro-nutrients |  |  |
| H_3_BO_3_ | 2.86 | 30 |
| MnCl_2_ 4H_2_O | 1.81 |  |
| ZnSO_4_ 7H_2_O | 0.22 |  |
| CuSO_4_ 5H_2_O | 0.08 |  |
| H_2_MoO_4_ H_2_O | 0.09 |  |

***Table S2.*** *Differences in total dry biomass, roots, stems, pods and leaves performed through a one-way ANOVA followed by Duncan test. Data refer to the HL treatment. Different letters indicate a significant difference.*

| Comparison of means | Total biomass | Roots | Stems | Pods | Leaves |
| --- | --- | --- | --- | --- | --- |
| WTNF | 129.309^a^ | 1.742^a^ | 1.642^a^ | 0.442^a^ | 2.417^a^ |
| WTF | 118.540^ab^ | 1.592^a^ | 1.500^a^ | 0.292^ab^ | 2.083^ab^ |
| MGNF | 111.931^b^ | 1.117^b^ | 1.225^b^ | 0.517^bc^ | 1.933^b^ |
| MGF | 98.427^c^ | 0.883^b^ | 1.125^b^ | 0.367^c^ | 1.500^c^ |

***Table S3.*** *Differences in net C uptake at different absorbed PPFD levels (Figure 2) performed through a one-way ANOVA followed by Duncan test. Data are means of five different days. Different letters indicate a significant difference.*

|  | **Low light** | | | | | | | | **High light** | | | |  |
| --- | --- | --- | --- | --- | --- | --- | --- | --- | --- | --- | --- | --- | --- |
| **PPFD** | **MGNF** | **MGF** | | **WTNF** | | | **WTF** | | **MGNF** | **MGF** | **WTNF** | **WTF** | |
| 0 | -1.886^a^ | -3.063^a^ | | -2.293^a^ | | | - | | -1.376^a^ | -1.219^a^ | -1.541^a^ | -1.368^a^ | |
| 50 | -1.313^a^ | - | | -1.412^a^ | | | 0.536^a^ | | - | - | - | - | |
| 100 | 2.201^ab^ | 0.579^b^ | | 1.033^b^ | | | 3.047^a^ | | -1.575^c^ | 1.067^b^ | -2.302^d^ | 1.943^a^ | |
| 150 | 4.965^ab^ | 3.051^b^ | | 3.463^b^ | | | 6.558^a^ | | -1.877^b^ | 0.837^a^ | -0.277^ab^ | 2.165^a^ | |
| 200 | 6.973^ab^ | 6.698^ab^ | | 6.269^b^ | | | 7.960^a^ | | 3.214^c^ | 5.387^b^ | 7.292^ab^ | 7.744^a^ | |
| 250 | 9.657^a^ | 8.635^a^ | | 8.064^a^ | | | 9.001^a^ | | -0.612^b^ | 6.450^a^ | 7.246^a^ | 8.472^a^ | |
| 300 | 11.447^a^ | 10.223^ab^ | | 9.608^b^ | | | 11.447^a^ | | 8.872^b^ | 8.434^b^ | 13.307^a^ | 11.543^a^ | |
| 350 | 12.786^ab^ | 12.792^ab^ | | 11.165^b^ | | | 14.431^a^ | | 9.489^b^ | 9.471^b^ | 12.615^a^ | 12.331^a^ | |
| 400 | 13.712^b^ | 12.034^c^ | | 15.582^a^ | | 14.726^ab^ | | | 12.225^b^ | 10.345^c^ | 18.421^a^ | 16.638^b^ | |
| 450 | 15.553^a^ | 14.806^a^ | | 16.312^a^ | | 15.874^a^ | | | 13.356^c^ | 12.584^c^ | 16.802^a^ | 15.371^b^ | |
| 500 |  | |  | |  | | |  | 14.492^b^ | 16.119^c^ | 17.039^a^ | 16.601^a^ | |
| 550 |  | |  | |  | | |  | 16.469^b^ | 14.396^c^ | 18.435^a^ | 17.573^a^ | |
| 600 |  | |  | |  | | |  | 17.419^b^ | 14.295^c^ | 19.242^a^ | 18.076^b^ | |
| 650 |  | |  | |  | | |  | 18.947^b^ | 14.726^c^ | 20.472^a^ | 18.289^b^ | |
| 700 |  | |  | |  | | |  | - | 15.943^c^ | 21.077^a^ | 17.870^b^ | |

***Table S4.*** *Differences in net C net CO2 exchange rate (NCER), evapotranspiration (E) and water use efficiency (WUE) in HL (Figure 3) per each experimental day performed through a one-way ANOVA followed by Duncan test. Different letters indicate a significant difference.*

|  | **NCER (g CO_2_ d^-1^)** | | | | **E (mmH_2_O d^-1^)** | | | | **WUE (g CO_2_/ mmH_2_O)** | | | |
| --- | --- | --- | --- | --- | --- | --- | --- | --- | --- | --- | --- | --- |
| **Day** | **MGNF** | **MGF** | **WTNF** | **WTF** | **MGNF** | **MGF** | **WTNF** | **WTF** | **MGNF** | **MGF** | **WTNF** | **WTF** |
| 02/07 | 0.743^a^ | 0.810^a^ | 1.005^a^ | 0.815^a^ | 2.422^a^ | 2.267^a^ | 2.218^a^ | 2.293^a^ | 0.011^a^ | 0.009^a^ | 0.010^a^ | 0.010^a^ |
| 03/07 | 1.070^a^ | 1.026^a^ | 1.186^a^ | 1.111^a^ | 2.816^a^ | 2.626^a^ | 2.639^a^ | 2.665^a^ | 0.011^a^ | 0.009^b^ | 0.010^ab^ | 0.010^ab^ |
| 04/07 | 1.502^a^ | 1.524^a^ | 1.663^a^ | 1.550^a^ | 3.199^a^ | 3.092^a^ | 2.951^a^ | 3.062^a^ | 0.013^a^ | 0.011^c^ | 0.012^b^ | 0.012^b^ |
| 05/07 | 1.488^ab^ | 1.465^a^ | 1.775^b^ | 1.591^ab^ | 3.229^a^ | 3.112^a^ | 2.971^a^ | 3.102^a^ | 0.012^a^ | 0.010^b^ | 0.013^a^ | 0.012^a^ |
| 06/07 | 1.585^ab^ | 1.470^a^ | 1.920^c^ | 1.779^bc^ | 2.894^a^ | 2.771^a^ | 2.716^a^ | 2.848^a^ | 0.012^a^ | 0.010^b^ | 0.013^a^ | 0.012^a^ |
| 07/07 | 2.381^ab^ | 2.157^a^ | 2.703^b^ | 2.407^ab^ | 3.461^a^ | 3.310^a^ | 3.229^a^ | 3.308^a^ | 0.014^ab^ | 0.012^c^ | 0.015^a^ | 0.014^b^ |
| 08/07 | 2.513^b^ | 2.110^a^ | 2.885^c^ | 2.544^b^ | 3.090^a^ | 2.978^a^ | 2.957^a^ | 2.950^a^ | 0.012^c^ | 0.011^d^ | 0.016^a^ | 0.014^b^ |
| 09/07 | 3.526^b^ | 2.977^a^ | 3.998^c^ | 3.527^b^ | 3.379^a^ | 3.295^a^ | 3.347^a^ | 3.239^a^ | 0.018^b^ | 0.015^c^ | 0.021^a^ | 0.019^ab^ |
| 10/07 | 3.674^b^ | 3.040^a^ | 4.187^c^ | 3.753^b^ | 3.663^a^ | 3.579^a^ | 3.642^a^ | 3.511^a^ | 0.019^b^ | 0.017^c^ | 0.023^a^ | 0.022^a^ |
| 11/07 | 4.103^b^ | 3.483^a^ | 5.015^d^ | 4.530^c^ | 3.444^a^ | 3.402^a^ | 3.451^a^ | 3.455^a^ | 0.024^b^ | 0.021^c^ | 0.029^a^ | 0.027^a^ |
| 12/07 | 4.948^b^ | 4.232^a^ | 5.770^c^ | 5.287^b^ | 4.308^a^ | 4.223^a^ | 4.203^a^ | 4.060^a^ | 0.022^b^ | 0.020^c^ | 0.027^a^ | 0.026^a^ |
| 13/07 | 5.518^b^ | 4.447^a^ | 5.992^c^ | 5.552^bc^ | 3.024^a^ | 2.923^ab^ | 2.879^ab^ | 2.757^b^ | 0.016^a^ | 0.010^a^ | 0.013^a^ | 0.014^a^ |
| 14/07 | 5.276^b^ | 4.415^a^ | 5.786^b^ | 5.516^b^ | 4.124^a^ | 3.991^a^ | 3.864^a^ | 3.758^a^ | 0.025^b^ | 0.022^c^ | 0.030^a^ | 0.030^a^ |
| 15/07 | 5.000^b^ | 4.189^a^ | 4.641^c^ | 5.381^bc^ | 3.460^a^ | 3.341^a^ | 3.177^a^ | 3.116^a^ | 0.031^b^ | 0.027^c^ | 0.039^a^ | 0.037^a^ |
| 16/07 | 5.630^b^ | 4.658^a^ | 6.135^b^ | 6.032^b^ | 2.958^a^ | 2.870^a^ | 2.729^a^ | 2.703^a^ | 0.038^b^ | 0.032^c^ | 0.048^a^ | 0.046^a^ |
| 17/07 | 5.453^b^ | 4.698^a^ | 6.421^c^ | 6.294^c^ | 3.045^a^ | 2.918^a^ | 2.805^a^ | 2.754^a^ | 0.039^b^ | 0.034^c^ | 0.051^a^ | 0.048^a^ |
| 18/07 | 5.796^ab^ | 4.958^a^ | 6.863^c^ | 6.072^bc^ | 3.625^a^ | 3.504^ab^ | 3.328^ab^ | 3.238^b^ | 0.035^b^ | 0.030^c^ | 0.045^a^ | 0.043^a^ |
| 19/07 | 5.902^ab^ | 5.266^a^ | 7.271^c^ | 6.460^b^ | 3.696^a^ | 3.581^ab^ | 3.401^ab^ | 3.323^b^ | 0.033^b^ | 0.029^c^ | 0.043^a^ | 0.041^a^ |
| 20/07 | 6.239^ab^ | 5.539^a^ | 7.358^c^ | 6.708^bc^ | 3.441^a^ | 3.355^ab^ | 3.079^b^ | 3.026^b^ | 0.041^c^ | 0.036^d^ | 0.055^a^ | 0.050^b^ |
| 21/07 | 6.295^ab^ | 5.596^a^ | 7.466^b^ | 6.720^ab^ | 3.055^a^ | 3.002^ab^ | 2.734^ab^ | 2.680^b^ | 0.046^c^ | 0.041^d^ | 0.063^a^ | 0.056^b^ |
| 22/07 | 6.644^a^ | 6.031^a^ | 8.058^b^ | 7.171^ab^ | 3.227^a^ | 3.223^a^ | 2.980^a^ | 2.924^a^ | 0.046^c^ | 0.042^c^ | 0.062^a^ | 0.056^b^ |
| 23/07 | 6.923^ab^ | 6.380^a^ | 7.660^b^ | 7.136^ab^ | 3.626^a^ | 3.662^a^ | 3.058^b^ | 3.117^b^ | 0.044^c^ | 0.040^c^ | 0.061^a^ | 0.055^b^ |
| 24/07 | 6.929^ab^ | 6.384^a^ | 7.785^b^ | 7.203^ab^ | 3.269^a^ | 3.325^a^ | 2.704^b^ | 2.771^b^ | 0.048^c^ | 0.044^c^ | 0.065^a^ | 0.059^b^ |
| 25/07 | 6.663^ab^ | 6.134^a^ | 7.401^b^ | 6.701^ab^ | 3.477^a^ | 3.534^a^ | 2.820^b^ | 2.874^b^ | 0.043^b^ | 0.040^b^ | 0.059^a^ | 0.053^a^ |
| 26/07 | 6.427^ab^ | 6.048^a^ | 7.363^b^ | 6.772^ab^ | 3.788^a^ | 3.864^a^ | 3.106^b^ | 3.232^b^ | 0.042^c^ | 0.038^c^ | 0.058^a^ | 0.051^b^ |

***Table S5.*** *Differences in* *Fv/Fm values between Eiko and MinnGold calculated in low and high light. The values are means ± deviation standard (n=6 in low light and n=3 in high light). Different letters indicate a significant difference between the two varieties (p<0.05). In this case, no significant difference was found.*

|  | MinnGold | Eiko | p-value |
| --- | --- | --- | --- |
| Low Light | 0.812 ± 0.010^a^ | 0.825 ± 0.012^a^ | 0.61 |
| High Light | 0.775 ± 0.241^a^ | 0.934 ± 0.112^a^ | 0.38 |

**Figures**

***Figure S1.*** *Leaf light curves for Eiko (dark green dots) and MinnGold (light green dots). Leaf were taken from plants grown in HL treatment. Vertical bars indicate the standard error (n=3).*

**
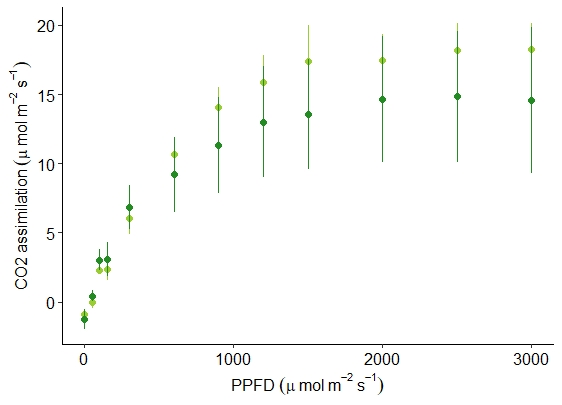
**

***Figure S2.*** *Air temperature (°C) and vapour pressure deficit (vpd, kPA) during the HL experiment for MinnGold (light green) and Eiko (dark green) in non-fluctuating and fluctuating light conditions.*


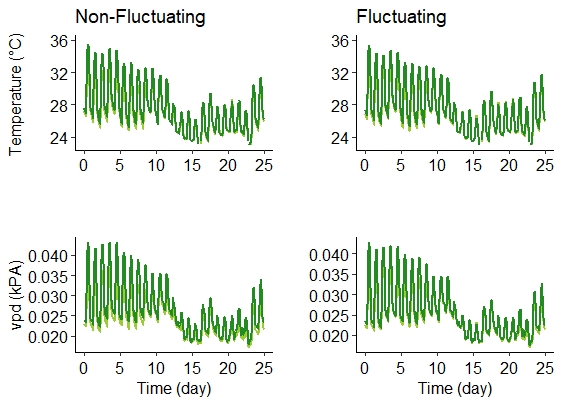


***Figure S3.*** *Net primary production (NPP) vs hourly PPFD on three different days (9,16 and 28 days after germination) for MinnGold (light green) and Eiko (dark green). The data are taken from 4 am to 7 pm under non-fluctuating light in the HL. The arrows indicate the direction of the loop, starting from the morning till the afternoon.*

***
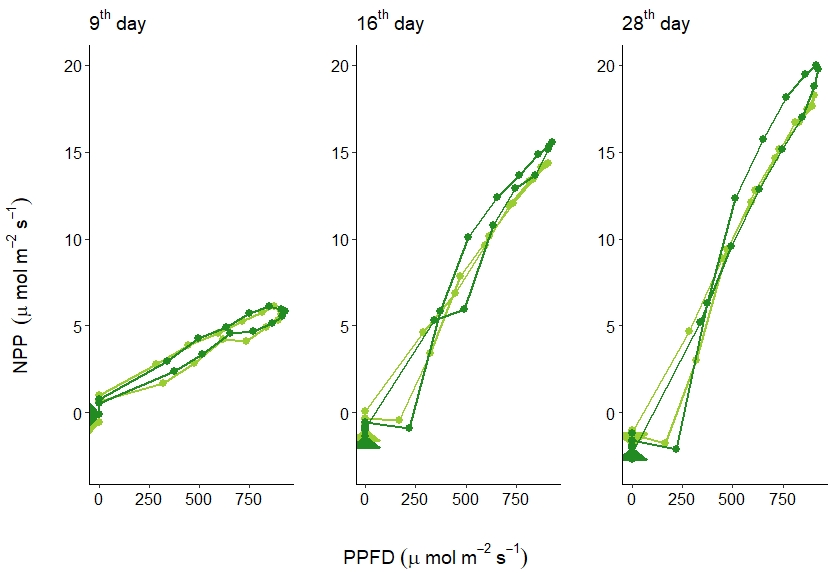
***

***Figure S4.*** *Changes in transmitted light (normalized to the minimum) for MinnGold (light green line) and Eiko (dark green line). The data are taken in the first hour of the morning from 6 am to 7 am under non-fluctuating light on the last day of the experiment. The time to reach steady state (τ) has been calculated as for the induction experiments (see Methods), the p-value is the result of the t-test.*

***
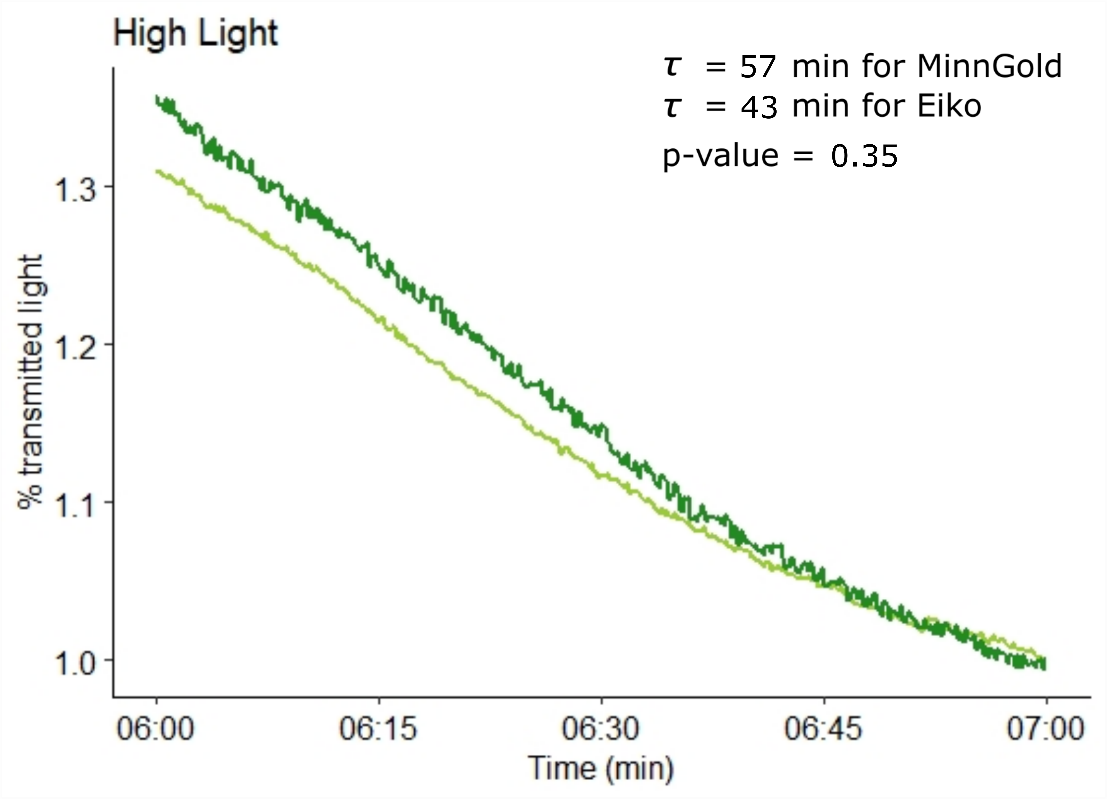
***

***Figure S5.*** *NPQ calculated in Low and High light for Eiko and Minngold during light fluctuations.*

***
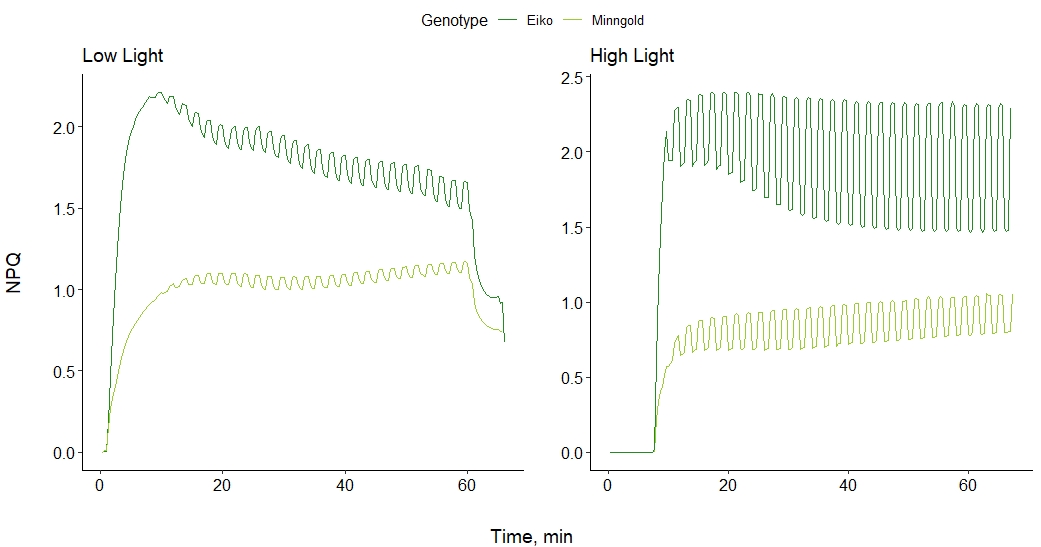
***
